# Acquired factor VIII deficiency: two case reports and a review of literature

**DOI:** 10.1186/s40164-017-0068-3

**Published:** 2017-03-24

**Authors:** Lan Mo, George C. Bao

**Affiliations:** Weill Cornell Medicine, New York-Presbyterian Lower Manhattan Hospital, 170 William Street, New York, NY 10038 USA

**Keywords:** Acquired factor VIII deficiency, Acquired hemophilia A, Parvovirus B19, Myositis, Immunosuppression, Glucocorticoids, Intravenous Immunoglobulin, Rituximab

## Abstract

**Background:**

Acquired factor VIII (FVIII) deficiency, or acquired hemophilia A (AHA), is a rare autoimmune disorder involving antibody-mediated depletion of coagulation FVIII, leading to severe, life-threatening bleeding. The condition is often associated with other autoimmune disorders, and its treatment involves replacement of FVIII and various modes of immunosuppression. Recently, a few noteworthy therapeutic advances have been made. We present two cases of severe AHA in Chinese women. One of these women developed this disorder in the setting of possible parvovirus B19 infection, which has not yet been reported in association with AHA. Other notable features of her case included paradoxical venous thrombosis and possible association with Sjogren’s syndrome and myositis. The other woman failed to respond to usual first-line therapies despite exhibiting a less severe clinical course, illustrating the varied but potentially stubborn behavior of this disorder.

**Case 1:**

An 87-year-old woman presented with diffuse ecchymoses, melena, vaginal bleeding. Labs showed hemoglobin (Hgb) nadir of 5.7 mg/dL, elevated partial thromboplastin time (PTT), FVIII level <1%, mixing study consistent with an inhibitor, elevated anti-Sjogren’s-Syndrome-related antigen A antibody, elevated creatinine kinase, and elevated parvovirus IgM and IgG. Imaging of her arm showed diffuse myositis and deep venous thrombosis. After intravenous and oral steroids, her FVIII levels normalized, and her symptoms subsided.

**Case 2:**

A 59-year-old woman presented with recurrent ecchymoses and hematomas in her extremities. Labs showed Hgb of 11.7 mg/dL, elevated PTT, FVIII level of 3%, and mixing study consistent with an inhibitor. Despite receiving a long course of steroids, several courses of IVIG, and a few courses of Rituximab, her FVIII level remained critically low.

**Conclusion:**

The rarity of AHA limits our understanding of this disease and the ability to perform trials to discover optimal therapies. We hope that these case reports and discussion will shed further light on the varied clinical manifestations and natural histories of this disorder to guide better recognition and treatment of AHA.

## Background

Factor VIII (FVIII) is an important protein in the coagulation cascade. Produced in the liver, it is normally bound to and stabilized by von Willebrand factor in the circulation. When activated by thrombin, it binds to factor IX to form a “tenase complex” that activates factor X [22]. Patients who lack FVIII suffer from hemophilia A. The disease most often occurs in its congenital form, but individuals may also acquire hemophilia A through an autoimmune process, in which an inhibitor antibody depletes FVIII. This can result in severe, spontaneous, life-threatening bleeding. Among the various acquired coagulation factor deficiencies involving an inhibitor, FVIII deficiency is the most prevalent. Yet, the disorder is still remarkably rare, and given its rarity, our understanding of it remains limited despite knowledge of it for several decades. We present two cases of acquired hemophilia A (AHA) in Chinese women. We will also review AHA, its epidemiology, risk factors, natural history, and the latest research concerning its management.

## Case 1

An 87-year-old Chinese woman with history of mild anemia and diabetes type 2 was admitted after falling at home. Her emergency physician found diffuse ecchymoses of her right cheek. She had pain in her bilateral arms and legs. Labs showed hemoglobin of 7.4 mg/dL, acute kidney injury, and rhabdomyolysis. Her PTT was >150 s, which did not correct in subsequent mixing studies, indicating the presence of an inhibitor. PT and INR were normal. FVIII was measured at <1% with inhibitor titer of 27.6 Bethesda units (BU).

During her hospital stay, she developed melena and vaginal bleeding, and her hemoglobin dipped to 5.7 mg/dL. An esophagogastroduodenoscopy showed multiple duodenal ulcers, treated with epinephrine injections and clipping. In total, she received 7 units of packed red blood cells and 3 units of fresh frozen plasma. Additional positive laboratory findings included lupus anticoagulant, anti-nuclear antibody (ANA) at 1:160, and anti-Sjogren’s-Syndrome-related antigen A (anti-SSA) antibody. The erythrocyte sedimentation rate was 109 mm/h, and the C-reactive protein was 25.26 mg/dL. Given concern for aplastic anemia initially, the team had also checked serologies for parvovirus and found positive IgM and IgG antibodies. When rechecked two weeks later, the parvovirus IgM antibody level normalized, while the IgG antibody remained elevated.

The patient’s creatinine kinase (CK) peaked at 7587 U/L, and her myoglobin peaked at 1366 ng/mL. Because of pain and swelling in her bilateral arms, we performed a CT of her arms, which showed edema and inflammation of muscle groups in both forearms. Incidentally, the CT also showed third spacing involving the subcutaneous soft tissues of both flanks, findings that were consistent with myositis.

While the patient’s arterial duplex study was negative for clot, the venous duplex was significant for superficial venous thromboses in both cephalic veins and right upper extremity deep venous thrombosis in the brachial and radial veins. Given her bleeding risk, no anticoagulation was started, and an inferior vena cava catheter was placed.

Her edema, pain, and CK markedly improved after the initiation of intravenous steroids. Unfortunately, due to her extensive upper extremity edema, she developed compressive neuropathy of her bilateral median and ulnar nerves, confirmed on an electromyogram and nerve conduction study, leading to functional disability in her hands.

Though initially she was suspected of having Sjogren’s given her positive anti-SSA along with other symptoms including xerostomia and arthralgias, her rheumatologist ultimately designated her condition as undifferentiated connective tissue disease, and she was started on hydroxychloroquine. A biopsy of her endometrium, performed because of her earlier vaginal bleeding, was negative for malignancy. Her symptoms ultimately were subdued after a long course of prednisone tapered over several months, and her FVIII level recovered to 180% on a repeat measurement 3 months later.

## Case 2

A 59-year-old Chinese woman with gout developed upper extremity bruising and a large, left calf hematoma after using topical herbs containing Corydalis turtschaninovii, *Salvia miltiorrhiza* Bge, and largehead Atractylodes rhizome to treat alopecia she had been experiencing for a year. Initial evaluation revealed a prolonged PTT, and subsequent labs revealed a critically low FVIII activity of 3%, an inconclusive mixing study, and a negative lupus anticoagulant. Based on her clinical symptoms and these laboratory findings, she was hospitalized, at which time her Hgb was 11.7 mg/dL and PTT was 66.7 s. A rheumatologic laboratory panel was negative. Given concern for AHA, she was treated empirically with high dose methylprednisolone at 1 mg/kg and a dose of desmopressin. Later, she received two doses of recombinant FVIII and was discharged on a prednisone taper.

Though her calf hematoma resolved, she then developed a left thigh hematoma a month later. To address this, she again received recombinant FVIII. Worsening of her bruising followed, and she was again admitted, this time receiving a 3-day course of intravenous immunoglobulin (IVIG), while also having her prednisone dose increased. A bone marrow biopsy was negative for malignancy. Her hematoma improved, and her prednisone continued to be tapered. An inhibitor titer of 22.4 BU was then found.

A month afterward, she developed recurrent hematomas in her legs and oral bleeding. She was again admitted for a second course of IVIG, while her prednisone dose was increased again. Following this treatment, though her hematoma improved, she developed livedo reticularis, thought at the time to be a reflection of underlying, undiagnosed connective tissue disease. Her leg hematoma did not improve until she received a third course of IVIG. Yet another month later, she developed another large, right leg hematoma, and was prescribed her fourth course of IVIG.

Throughout this entire course of events, her FVIII levels did not improve, and her PTT never fully normalized. Consequently, she was started on a trial of rituximab, to which she did not respond, so another round of rituximab was given. At this point, prednisone had been tapered off. During her second rituximab course, she developed shingles on her right chest, which resolved with valacyclovir. To date, she still has a low FVIII activity of 8%, though she has not had any recent bleeding episodes. She has stopped using her topical herbs.

## Discussion

### Pathophysiology, epidemiology, and risk factors

Acquired FVIII deficiency is an autoimmune disorder mediated by polyclonal IgG antibodies, some of which have proteolytic properties, capable of hydrolyzing FVIII into smaller fragments. The disorder is thought to arise from the adaptive immune system, wherein FVIII antigen is endocytosed by antigen presenting cells, which ultimately stimulate B cells to generate FVIII-specific antibodies. Of note, normal, healthy individuals possess endogenous antibodies to FVIII, suggesting that immune tolerance mechanisms, such as in the form of regulatory T cells, keep such antibodies at bay. The autoimmune disorder is thought to emerge when such immune tolerance mechanisms break down [23].

About a third of patients with congenital hemophilia A develop inhibitory antibodies when treated with FVIII replacement, but such inhibitors arise very rarely in people without pre-existing hemophilia, at a reported incidence of 1.48 or 1.34 per million/year in two UK studies [9]. The age of presentation is bimodal; a significant portion of disease emerges in the postpartum setting, while most incidence of disease arises in patients over the age of 50. As with many diseases, age portends a worse prognosis. There is no apparent stronger predilection for men or women. This disorder occurs in the postpartum setting, in those with connective tissue disease, as a paraneoplastic syndrome, and after the use of certain medications such as penicillin, sulfamides, and phenytoin [12]. Rare, ambiguous associations have been reported following trauma or even minor surgical procedures. However, in about half of cases, no cause is identified.

Some herbal medications have been known to be associated with autoimmunity, but no associations between AHA and herbal medications have been reported [19]. A few herbal medications may potentiate bleeding, and *Salvia miltiorrhiza*, which our second patient used, has been known to interfere with hemostasis through several mechanisms, including mimicking antithrombin III [5, 15]. However, neither Corydalis turtschaninovii nor largehead Atractylodes rhizome, which this patient also used, is known to be associated with autoimmunity or bleeding.

No study to date exists reporting the incidence of acquired FVIII inhibitors across ethnic groups. A study looking at six hemophilia centers in China, encompassing 1435 patients with congenital hemophilia, suggested that the prevalence of acquired inhibitors in Chinese hemophiliacs is lower than that reported for other ethnic groups, at 3.9% compared to 5–7% in non-Chinese [33]. To date, the largest retrospective study looking at AHA in the Chinese population, encompassing 49 patients at a single center in China over the course of 18 years, revealed that the risk factors and clinical manifestations are similar to patients studied in non-Chinese populations [34]. Similarly, a retrospective study looking of 65 Taiwanese patients with AHA also found that those patients shared similar risk factors to non-Asians [16]. Another systematic review of 111 patients at a Thai university medical center database suggested that the age of presentation might be younger in Asian patients [4].

In its association with connective tissue disorders, AHA most commonly occurs in the context of rheumatoid arthritis and systematic lupus erythematosis. There have been a few case reports of the disease arising in Sjogren’s syndrome [32]. Furthermore, there is not a strong link between this disorder and myositis, though the disorder has been found in association with dermatomyositis [1].

Thus far, no linkage has been made between parvovirus B19 infection and acquired FVIII inhibitor. There are several case reports of parvovirus B19 infection causing myositis in adults, but, in one case series, muscle biopsies in patients with myositis and parvovirus infection did not reveal the presence of B19 DNA, suggesting that parvovirus is not directly responsible for causing the myositis [6, 27]. Nevertheless, there may be an indirect immunologic mechanism involved, as parvovirus infection has been thought to precipitate several autoimmune disorders, most famously rheumatoid arthritis and systemic lupus erythematosis. Parovirus infection has also been associated with antiphospholipid syndrome, adult-onset Still’s disease, dermatomyositis, Sjogren’s syndrome, scleroderma, temporal arteritis, Henoch-Schonlein purpura, granulomatosis with polyangiitis, Kawasaki disease, and polyarteritis nodosa. Such autoimmunity may occur through autoantibody generation due to epitope similarity between viral and human antigens [28].

### Clinical manifestations, diagnosis, and prognosis

Patients with hemophilia A often present with abnormal or spontaneous, unexpected bleeding. In contrast to congenital hemophilia, which is famous for causing hemarthrosis, AHA patients most often present with large, sometimes life or limb-threatening, soft tissue or subcutaneous bleeding, manifesting as ecchymosis. Muscle, gastrointestinal, vaginal, and joint bleeding follow in frequency. Often, patients experience multiple sites of bleeding [8].

It is unclear how often deep venous thrombosis occurs concomitantly with AHA, but the phenomenon is thought to be extremely rare. Several case reports of deep venous thrombosis occurring simultaneously with both acquired hemophilia A and B exist [10, 29]. One proposed mechanism to explain the paradoxical occurrence of both is that the tissue factor–factor VIIa complex of the extrinsic coagulation pathway, which normally activates factor X via a factor XI and factor VIII-dependent route, can, in sufficient quantities, activate factor X directly, leading to the formation of a clot. AHA is often associated with widespread vascular injury, thus generating high quantities of tissue factor [10]. In the general hemophiliac population, the most frequent cause of thrombosis is the administration of the thrombogenic bypassing agents activated prothrombin complex concentrate (aPCC) and recombinant factor VIIa (rVIIa), though our patient who developed the DVT did not receive either of these products [13].

Acquired hemophilia A should be suspected when a patient’s PTT is prolonged. Afterward, a search for an inhibitory antibody should be performed through a mixing study in which the patient’s serum is mixed with normal serum. If the PTT prolongation is simply due to a factor deficiency, then the PTT should correct after mixing. If an inhibitor to FVIII exists, the PTT will remain prolonged even after mixing. The Bethesda Unit (BU), obtained when FVIII activity is measured while the patient’s plasma is incubated with normal plasma, is a measure of inhibitor titer.

Aside from bleeding, the greatest cause of mortality is actually from sepsis that may arise when attempts are made to eliminate the inhibitor through immunosuppressive therapies. Survival is best when the patient is able to achieve complete remission, a state that has slightly varying definitions across different studies, but is defined by one study as undetectable inhibitor and FVIII more than 70 IU/dL when immunosuppression is stopped [11].

### Management strategies

The two goals in the treatment of AHA are 1) to control active bleeding and 2) to eliminate the inhibitor.

The methods to control bleeding are hemostatic control and the use of bypassing agents, both of which should be employed concomitantly given that conventional methods of local hemostatic control are often insufficient and do not prevent a recurrence of bleeding. For non-severe bleeding, the use of desmopressin, which causes a release of stored FVIII and von Willebrand factor from endothelial cells, is an option. Though the available evidence suggests that the response to desmopressin is unpredictable, it is more readily available at many hospitals compared to other therapies and is thus often employed. For severe bleeding but with low titers (<5 BU), the use of desmopressin and recombinant human recombinant FVIII product may be effective [17].

At inhibitor titer levels >5 BU, replacement with recombinant factor VIII is generally ineffective. For severe bleeding in these patients, use of bypassing agents, which include activated prothrombin complex concentrate (aPCC; e.g., factor VIII inhibitor bypassing activity [FEIBA]) or recombinant human factor VIIa (rVIIa; e.g., NovoSeven), is recommended. Thus far, in the congenital hemophiliac population, studies have not conclusively shown whether rVIIa or aPCC is more effective than the other [18]. In the largest longitudinal, prospective, observational study to date for acquired hemophiliacs (from the European Acquired Hemophilia Registry, EACH2), both aPCC and rVIIa demonstrated similar efficacy in stopping bleeding (93.0%; P = 1) [2]. Certain patients may respond better to one or the other, and the decision to switch from one to the other is made on a case-by-case basis with close clinical monitoring. Often, multiple, sequential, alternating infusions of both bypassing agents are needed to control a bleeding episode.

Porcine recombinant factor VIIIa (rVIIIa) may work better than human-derived rVIIIa because inhibitors are less likely to recognize it [17, 35]. Hyate:C was a plasma-derived porcine factor VIIIa that was associated with allergic reactions and transient thrombocytopenia and was discontinued in 2004 due to concerns that it was infecting recipients with porcine parvovirus [30]. In 2014, the US Food and Drug Administration approved a new recombinant porcine factor VIII (Obizur), which is expected to have a superior safety profile compared to Hyate:C while delivering the same efficacy [20, 24]. However, the cross-reactivity of anti-human FVIII antibody to porcine rVIIIa remains around 30–50% [30].

Much of present research aims to develop longer half-life clotting factors whose efficacy will remain longer in the body after administration. Achieving this involves conjugation of clotting factors with compounds such as polyethylene glycol (PEGylation), the fragment crystallizable (Fc) region of human antibodies, and recombinant albumin [3, 25]. While such advancements will assist in treating patients with acquired hemophilia, they will have the greatest benefit in those with hereditary hemophilia who currently require frequent, prophylactic clotting factor infusions.

Other novel therapeutic approaches depart from the paradigm of clotting factor replacement altogether and look for ways to bypass the deficiency of FVIII. Such methods include utilizing small inhibitory RNAs to disrupt the liver’s generation of antithrombin III, which inhibits several factors in the intrinsic coagulation pathway, or utilizing an antibody (Concizumab) that binds tissue factor pathway inhibitor, which inhibits factor Xa [3]. Recently, a Japanese trial testing Emicizumab (ACE910), a bispecific antibody and FVIII mimetic, showed that this agent was effective in stopping bleeding in hereditary hemophilia A patients with high inhibitor titers. Emicizumab acts by bringing activated factor IX and factor X together, mediating the activation of factor X and thus essentially mimicking the function of FVIII. Given the 3-week half-life and high bioavailability of this subcutaneously administered drug, weekly administrations are possible, reducing the burden of frequent treatments for patients [31].

To eliminate the inhibitor, several options are available, and, thus far, research has not concluded that one method is superior to another. Given the rarity of this disease, it is difficult to design prospective randomized trials to explore and compare the efficacy of various treatment options. Glucocorticoids, cyclophosphamide, rituximab, or a combination of these agents have all shown efficacy. To date, the largest longitudinal prospective, observational study of 331 patients comparing glucocorticoids, glucocorticoids with cyclophosphamide, and rituximab-based regimens found that glucocorticoids with cyclophosphamide led to the highest rate of complete remission. However, none of the three therapeutic strategies had a significant effect on survival in the median 262 days of follow-up. Furthermore, patients who began therapy with higher inhibitor titers and lower FVIII levels were less likely to achieve stable remission [7].

Toxic effects are common in all three therapeutic routes, though the combination of steroids and cyclophosphamide seems to confer the highest risk of neutropenia. This is something to be mindful of in the already relatively immunocompromised elderly population. Cyclophosphamide is also procarcinogenic at high doses and poses a risk of malignancy, making it sometimes prudent to avoid this drug in younger patients [36]. Given its relatively lower risk and severity of toxicity and easier route of administration, steroids are often the preferred first line therapy.

Other eradication strategies include the use of intravenous immunoglobulin, though this method often requires multiple treatment sessions, and the results are often disappointing in those with high inhibitor titers [11]. Anecdotal success has also been noted with cyclosporine, cladribine, tacrolimus, mycophenolate mofetil, and plasmapheresis.

A few small trials have tested immune tolerance induction, in which FVIII is given to patients, sometimes in conjunction with an immunosuppressive agent such as cyclophosphamide, yielding promising results. The rationale for this treatment strategy lies in the theory that FVIII will stimulate B cells generating the inhibitor to proliferate, thus priming these cells to the effects of immunosuppressives. One study of 14 patients with AHA did imply that this technique could allow complete remission to be achieved earlier compared to simply utilizing immunosuppressives [26]. However, strong conclusions cannot yet be drawn from the small amount of available data [11]. Furthermore, most of the studies on immune tolerance showing promising results have been done on patients with congenital hemophilia.

Immunoadsorption is another technique that has been used with good results in some small trials and individual cases. The advantage of immunoadsorption over plasmapharesis is the specific removal of the inhibitor without removal of other clotting factors. In one cohort of 35 patients with AHA, immunoadsorption was used as an adjunct therapy to FVIII replacement, steroids, IVIG, and cyclophosphamide [36]. In this trial, rapid cessation of bleeding and undetectable inhibitor counts were achieved in a median of 3 days, though the time to complete remission, defined as normalization of FVIII level and undetectable inhibitor level in the setting of no immunosuppression or factor replacement, was similar to other trials.

It is worth noting that given enough time, AHA may go away on its own. In a survey of 31 patients who received no therapy other than supportive transfusions and factor VIII concentrate, 11 patients experienced spontaneous remission in an average of 14 months [14]. Another single-institutional study followed for an average of 31 months (range of 4–120 months) 16 patients with AHA who did not receive immunosuppressive therapy. Five of these patients underwent spontaneous remission [21]. However, despite the possibility of spontaneous remission, the general consensus is that elimination therapy should be initiated as soon as possible.

## Conclusion

Acquired factor VIII deficiency is a bleeding disorder that requires prompt diagnosis and management to avert severe, life-threatening bleeding and death. Despite knowledge of this disorder of coagulation for several decades, relatively little is still known about this disease because of its rare incidence. Its rarity poses challenges in our ability to recognize and diagnose this disease as well as to perform scientific trials to discover better therapies. However, progress has been made in understanding this disease, and, as more case reports accumulate, we will be able to enhance our understanding and refine our therapeutic strategies.

## Abbreviations

AHA: acquired hemophilia A
FVIII: factor VIII
IVIG: intravenous immunoglobulin
BU: Bethesda units
aPCC: activated prothrombin complex concentrate
rVIIa: recombinant factor VIIa
rVIIIa: recombinant factor VIIIa
